# Using Explainable Artificial Intelligence to Obtain Efficient Seizure-Detection Models Based on Electroencephalography Signals

**DOI:** 10.3390/s23249871

**Published:** 2023-12-16

**Authors:** Jusciaane Chacon Vieira, Luiz Affonso Guedes, Mailson Ribeiro Santos, Ignacio Sanchez-Gendriz

**Affiliations:** Department of Computer Engineering and Automation—DCA, Federal University of Rio Grande do Norte—UFRN, Natal 59078-900, RN, Brazil; affonso@dca.ufrn.br (L.A.G.); mailsonribeiro@ufrn.edu.br (M.R.S.); ignaciogendriz@dca.ufrn.br (I.S.-G.)

**Keywords:** machine learning, Explainable AI, electroencephalography, epilepsy

## Abstract

Epilepsy is a condition that affects 50 million individuals globally, significantly impacting their quality of life. Epileptic seizures, a transient occurrence, are characterized by a spectrum of manifestations, including alterations in motor function and consciousness. These events impose restrictions on the daily lives of those affected, frequently resulting in social isolation and psychological distress. In response, numerous efforts have been directed towards the detection and prevention of epileptic seizures through EEG signal analysis, employing machine learning and deep learning methodologies. This study presents a methodology that reduces the number of features and channels required by simpler classifiers, leveraging Explainable Artificial Intelligence (XAI) for the detection of epileptic seizures. The proposed approach achieves performance metrics exceeding 95% in accuracy, precision, recall, and F1-score by utilizing merely six features and five channels in a temporal domain analysis, with a time window of 1 s. The model demonstrates robust generalization across the patient cohort included in the database, suggesting that feature reduction in simpler models—without resorting to deep learning—is adequate for seizure detection. The research underscores the potential for substantial reductions in the number of attributes and channels, advocating for the training of models with strategically selected electrodes, and thereby supporting the development of effective mobile applications for epileptic seizure detection.

## 1. Introduction

Epilepsy is a pathology that affects approximately 50 million individuals worldwide, with an estimated 2.4 million new cases developing annually. The prevalence of the disease varies, influenced by numerous factors, yet it is predominantly observed in developing countries. This trend underscores the importance of advancements in treatment and preventive measures as pivotal in curtailing such rates [1]. Moreover, individuals diagnosed with epilepsy often experience diminished quality of life, arising from social isolation, limitations in performing daily tasks, societal stigma, and psychological impacts on the patients and their families [2].

An epileptic seizure is a transient occurrence with diverse clinical manifestations that may affect sensory, motor, and autonomic functions; consciousness; memory; and can cause perceptual distortions [2]. Electroencephalography (EEG), a technique for measuring brain activity by recording electrical signals from the scalp, is commonly employed in the diagnosis of this condition. It can reveal the abnormally heightened synchrony of neuronal activity characteristic of epileptic seizures. However, it is not uncommon for patients with recurrent seizures to exhibit normal EEG patterns during and between seizure episodes [2], thus complicating accurate diagnosis.

Epileptic events known as absence seizures are particularly subtle, often marked by minimal motor activities [3]. Clinically identifying the onset and conclusion of such seizures poses a challenge, more so with atypical absence seizures where the indicators are less pronounced. Consequently, EEG signal monitoring becomes essential to the confirmation of these types of seizures [4].

The detection of epileptic seizures can be useful for recording and monitoring seizure frequencies in patients with the disease [5], such as those who experience absence seizures [6]. Simplified models for epileptic seizure detection—using few channels and requiring low computational costs—enable the implementation of embedded and wearable systems aimed at identifying seizures for subsequent medical analysis, as demonstrated in the study conducted by [6], where they achieved a sensitivity of 98.4% in the detection of typical absence seizures.

Given the significance and intricacy of seizure detection, considerable efforts have been directed towards machine learning (ML)-based methodologies for the automated analysis of EEG signals. Supervised ML classifiers are commonly employed in this domain [7,8,9,10,11,12], along with deep learning (DL) techniques [13,14]. These approaches are geared towards developing models adept at binary classification [7,8,9,11,12] as well as multiclass classification tasks [15,16].

Feature extraction is a pivotal element in enhancing the performance of ML models, particularly within the realm of EEG signal analysis. The methodologies for feature extraction in this context span across time, frequency, time–frequency, and non-linear domains  [17], utilizing values from EEG signals over brief temporal windows. Time domain techniques often employ statistical metrics such as standard deviation, kurtosis, skewness, and mean [8,15,16]. Frequency domain approaches typically involve the use of Fast Fourier Transform (FFT) or Discrete Wavelet Transform (DWT) coefficients, either directly or through derived statistical measures of these coefficients [7,9,10,11,12,16,18,19]. Owing to the inherent non-linearity of EEG signals [20], certain studies also focus on extracting non-linear features, including Sample Entropy, Wavelet Entropy [21], and the Lyapunov exponent [22].

In addition to feature extraction, feature selection is crucial for the development of accurate and efficient machine learning models [23]. Several studies have proposed feature extraction techniques aimed at reducing dimensionality, such as Principal Component Analysis (PCA) [9]. Nonetheless, many of these techniques do not account for the specific channels associated with the selected features, which can necessitate the use of numerous electrodes. This requirement potentially complicates the deployment of compact and embedded devices.

Explainable Artificial Intelligence (XAI) has recently emerged as a promising approach to elucidate the intricacies of model decision-making processes, offering insights into how specific outcomes are derived. Such transparency is indispensable in healthcare applications, ensuring that model behaviors can be understood and trusted by practitioners [24]. An example of an application is the work of [25], which used XAI techniques for predicting strokes through interpretable analysis of EEG signals.

Among the XAI approaches, the SHapley Additive exPlanations (SHAP) method, as presented in [26], provides significance values for each feature in predicting the data, as discussed in [24]. According to [27], where the author conducted a study to identify epileptic seizures through the analysis of EEG signals using the SHAP technique, interpreting the model’s output allows us to understand where it needs improvement. They mentioned three benefits of SHAP: global interpretability (contribution of each feature), local interpretability (each individual sample has its SHAP value), and versatility (usable with any tree-based model) [27].

In this study, we aimed to develop an efficient method for detecting epileptic seizures in EEG, focusing on reducing the number of channels used for signal acquisition and data processing. To achieve this, we applied an interpretable method—SHAP—to optimize machine learning models that perform binary classifications of EEG segments from interictal (period between epileptic seizures) and ictal (seizure period) states. Additionally, we also investigated the spatial proximity of relevant channels to the focal areas of epileptic seizures in the patients’ database.

The remainder of this work is organized as follows: Section 2 presents the concepts and operation of XAI and SHAP; Section 3 discusses related works; Section 4 describes the proposed approach; Section 5 presents the materials and methods, including the utilized dataset and experimental setup; Section 6 showcases the obtained results; Section 7 provides a discussion; and Section 8 highlights the main conclusions and suggests future work.

## 2. Explainable Artificial Intelligence

Advanced ML-based predictive models, including DL neural networks, can achieve excellent performance in mapping resources as the input to classes as the output of these models. Nonetheless, these models are often opaque, resembling ’black-box’ systems, which may inadvertently contribute to misinterpretations by neglecting data errors or human biases embedded within the training data, thereby affecting the decision-making process. Conversely, a transparent ML paradigm fosters the development of open and accountable models that are designed to address and mitigate such issues [28].

The explainability of an ML model is the elaboration of an interface between the human and the machine that is understandable to the human. Thus, the decision-making actions by the ML model need to be explainable [28].

Explainability transforms a non-interpretable model into one that can be interpreted, essentially delineating cause and effect relationships. Thus, Explainable Artificial Intelligence is designed to generate results that are readily understandable to users [29]. Consequently, such models bolster interpretability while preserving the precision of their predictions [30].

SHAP (SHapley Additive exPlanations) is a tool within the Python ecosystem that quantitatively attributes importance to individual features regarding their contribution to a model’s predictions, grounding its methodology in coalition game theory, where the feature values of an instance are treated analogously to players in a game [26,31,32].

**Figure 1:**
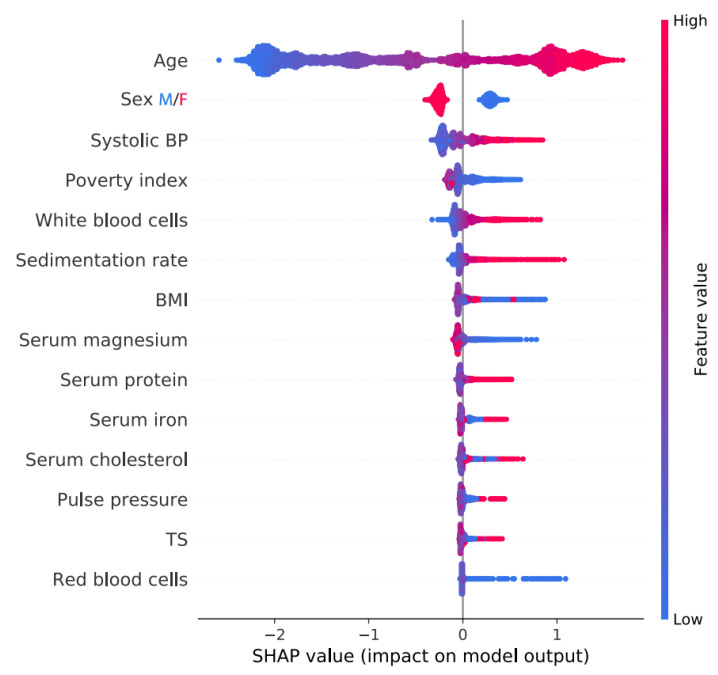
Shap summary plot [33].

One of the possible representations of SHAP values is the SHAP summary plot, in which features are ranked first by their global impact and then by points representing the SHAP values. Points that represent each feature of each sample in the graph are created—in blue, they represent low values of the contribution of the feature, and points colored in red represent high values, and there may be a color graduation between the points—which accumulate vertically, indicating the density [33]. Figure 1 shows this type of graph, in which the influence of certain attributes on mortality is observed. Thus, high SHAP values indicate, for a given attribute, a greater probability of death [33,34].

## 3. Related Works

The current landscape of EEG signal analysis covers various applications. There are works focused on brain–computer interfaces for controlling orthoses/prostheses [35,36,37], controlling computer systems and devices [38,39,40], muscular rehabilitation through neurofeedback [41,42], assessing psychological states and emotions [43,44], and diagnosing and treating neurological disorders [45,46], among various applications aimed at improving the quality of life of individuals, either in a functional or health context.

In the field of epileptic seizure identification, the landscape is extensive and diverse. Some works perform seizure identification through EEG signals, as presented in Table 1, while others use medical images [47,48,49]. Some works aim to detect only the presence or absence of a seizure, as shown in Table 1, while others aim to differentiate between seizure types [50,51,52], and some seek to predict seizures [53,54,55].

Literature reviews present a more comprehensive and comparative scenario. For instance, Ref. [17] discusses various feature extraction techniques in the time, frequency, time–frequency, and non-linear domains. Others highlight existing gaps in the field. Ref. [56] conducted a review on seizure prediction and reported that selecting the most significant channels is interesting for seizure prediction areas. Meanwhile, Ref. [57] conducted a bibliographic survey covering both seizure detection and prediction and concluded that channel selection is favorable for reducing computational costs, especially when the goal is to implement an online application. In their review work, Ref. [58] encourage the use of “non-black-box” classifiers as they can be more efficient when the objective is to find information about seizure localization. Ref. [59] reported some gaps in the research area, including the need to invest in a type of epilepsy known as absence seizures, due to the difficulty of visual identification [59].

In this study, a bibliographic search was conducted for publications that employed ML techniques (excluding DL) for the classification of binary data (interictal and ictal) in humans with epilepsy. It was chosen to use ML techniques because they are more feasible for performing model explainability analysis and observing the importance of features [60]. DL models, being “black-box”, make it difficult to understand predictions and are therefore less interpretable [61]. In addition, DL models are more prone to overfitting [62], have longer training times, and require a large amount of data [63].

Table 1 offers a comparative analysis of related works, succinctly summarizing the methodologies employed and the performance outcomes as measured by the metrics applied in each respective study. The datasets referenced in Table 1 originate from Temple University Hospital (TUH) [64] and the Children’s Hospital Boston–Massachusetts Institute of Technology (CHB-MIT) [65].

It is noticeable that works achieving around 99% accuracy require a high number of attributes, as is the case with 396 [12] and 1328 [18]. In the latter case, specificity was low compared with recall and accuracy, indicating that despite the large number of attributes, there was a challenge in achieving relative precision in identifying non-seizure data as non-seizure by the classifier.

Studies that applied methods to reduce the number of input vectors, such as Principal Component Analysis (PCA) and non-linear dimension reduction using t-distributed stochastic neighbor embedding (t-SNE), achieved precisions of 0.73 [9] and 0.47 [19], respectively. This result highlights the importance of selecting attributes using explainable methods.

Next, we provide a brief description of the works presented in Table 1.

Ref. [7] assessed various machine learning classifiers to distinguish between EEG records of patients with and without epileptic seizures. They utilized signal complexity measures and spectral power in different frequency bands for this purpose. In their findings, they indicated that the combination of complexity and spectral power achieved effective classification performance using a TUH dataset. However, in the study, they pointed out the need to consider gender differences as there is variation in EEG signals, which they intend to investigate in future work.

Ref. [8] developed a method for the automatic detection of generalized seizures in the TUH dataset by preprocessing EEG signals, extracting features in the time and frequency domains, and using machine learning classifiers, including Logistic Regression, Decision Tree, and SVM. SVM achieved the highest accuracy, reaching 0.93 in the binary classification of generalized seizures.

Ref. [9] used Logistic Regression as a classifier and extracted signal features through Fourier Analysis, with an emphasis on the energy distribution in different frequency bands. They also employed PCA and observed improvements in some performance metrics. The study involved 20 epilepsy patients and 20 healthy individuals. They suggested that proper feature selection before applying PCA is crucial to achieve significant improvements.

In [10], three wavelet-based feature extraction methods were applied and compared to classify multiple types of seizures in EEG data from the TUH database. Using the LightGBM classifier and without performing dimensionality reduction, they achieved a performance of approximately 0.84 in terms of weighted F1-score for the two classes analyzed.

Ref. [11] developed an automatic classification system for brain signals in multi-channel EEG records, using Wavelet Packet Decomposition to extract statistical features from frequency subbands such as mean absolute values, mean power, standard deviation, mean absolute value ratio, skewness, and kurtosis. Three Gradient Boosting Decision Tree-based classifiers were employed, with CatBoost achieving a classification accuracy of 0.88.

Ref. [12] developed an Automated Seizure Detection System using SVM and kNN classifiers and features extracted in the time, frequency, and time–frequency domains. The authors proposed generating metadata for the CHB-MIT database. Furthermore, they emphasized the importance of incorporating medical knowledge with machine learning methods.

Ref. [18] developed a hybrid seizure detection algorithm that combines continuous electroencephalography and integrated amplitude electroencephalography signals to diagnose epilepsy. They extracted features from multiple domains and performed classification using Random Forest. The study demonstrated high accuracy; however, they claim that the method is more suitable for longer seizures. They also developed a portable seizure detection system.

Ref. [19] employed a Random Forest classifier to select the most informative channels and a KNN classifier for the final discrimination between seizures and non-seizures. Feature extraction was based on the frequency domain of EEG signals, with 23 patients participating in the study. The combination of channel selection and non-linear dimensionality reduction enabled the method to achieve a recall performance of 0.81.

## 4. Proposed Approach

The proposal presented here for the efficient detection the epileptic seizures using EEG signals is composed of two phases. In the first one, the main contribution is the XAI-based approach to feature selection. Then, the second phase uses a similar procedure to obtain more efficient results. In both phases, the SHAP XAI Python library was used for feature selection.

**Figure 2:**
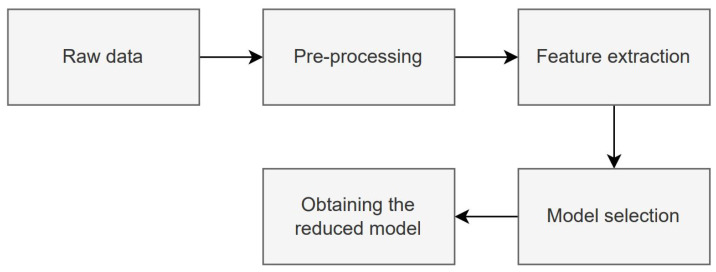
The first phase activity sequence of the proposed approach.

Figure 2 shows the activity sequence of the first phase, which is described as follows:

- Raw data: In this first part, the raw data are downloaded from the site and the training and test data indicated in the dataset are used.
- Pre-processing: The initial step in pre-processing involves segregating the data into two distinct classes: interictal (non-seizure) and ictal (encompassing various seizure types). For the purpose of binary classification, all seizure types are amalgamated into a single ’ictal’ class. Subsequently, data normalization is carried out followed by signal segmentation within a specified time window.
- Feature extraction: The features are extracted in the time domain for each of the channels of the segmented signal.
- Model selection: Training and testing with different ML models are performed. The ML model with the best performance in accuracy is chosen to be used in the next steps.
- Obtaining the reduced model: Based on the model selected in the previous phase, attribute selection is performed, considering the attributes that contributed the most to the prediction according to the SHAP value. New models with a reduced number of attributes are then trained.

The second phase of the proposed methodology was dedicated to diminishing the quantity of input vectors by amalgamating the most recurrently significant channels and features. This phase encompassed two procedures: feature selection followed by obtaining the reduced model.

- Feature selection: a combination is made with the most recurrent channels and features (product of the number of most recurrent channels and the number of most recurrent features) among the attributes that contributed most to the prediction according to the SHAP value.
- Obtaining the reduced model: A model is trained with the attributes from the combination in the previous step. The SHAP value is used again to rank the attributes that contributed most to the prediction, and then new models with a reduced number of attributes are trained, following the order of relevance.

## 5. Materials and Methods

In this section, the materials and methods used are presented, including a description of the chosen dataset and experimental setup.

### 5.1. Dataset

The database selection for the method implementation followed several criteria: it had to be a public database; contain data from humans with epilepsy; use surface EEG channels; include interictal and ictal segments; have at least the channels arranged in the international 10-20 system; be acquired at a sampling rate between 200 and 512 Hz, as it is the ideal range for observing epileptic data information [3]; include data from at least two patients; and provide information about the type of seizures and the brain location of each patient.

The database employed in this research is the publicly accessible dataset from the University of Beirut Medical Center (UBMC) [66]. This dataset comprises recordings from six epilepsy patients. The data were captured at a sampling rate of 500 Hz, encompassing in excess of 7 h of ictal recordings—including Complex Partial Seizure, Electrographic Seizures, and Video-detected Seizures without observable EEG changes—and over 7 h of interictal recordings [66]. Recordings were conducted using twenty-one surface electrodes aligned with the international 10-20 system for electrode placement [67].

Data were selected so that analyses were performed in two classes (interictal and ictal). In addition, of the 21 available channels, only 19 were used because there were missing records of two channels (Cz and Pz) in some recordings [66].

The documentation also contains information about the type of seizure for each patient, which are electrographic or Complex Partial Seizure. The focal points were also recorded, including Fp2, F4, F8, T6, Cz, C3, C4, T3, T3-P3, T3-C3, right temporal, left hemisphere, posterior temporal, fronto-temporal, diffuse onset, and no change over surface EEG [66].

The provided dataset is pre-labeled for training and testing across four classes and is available in the .mat file format. It encompasses 3,505,500 data points designated for training and 389,500 for testing, resulting from signal acquisition with a sampling rate of 500 Hz [66].

Feature extraction was performed using a window of 1 s (500 samples per second); thus, the number of entries was reduced to 7011 samples of training data (3479 samples of interictal data and 3532 samples of all classes of ictal data) and 779 test data samples (416 interictal data samples and 363 samples from all types of ictal data).

**Figure 3:**
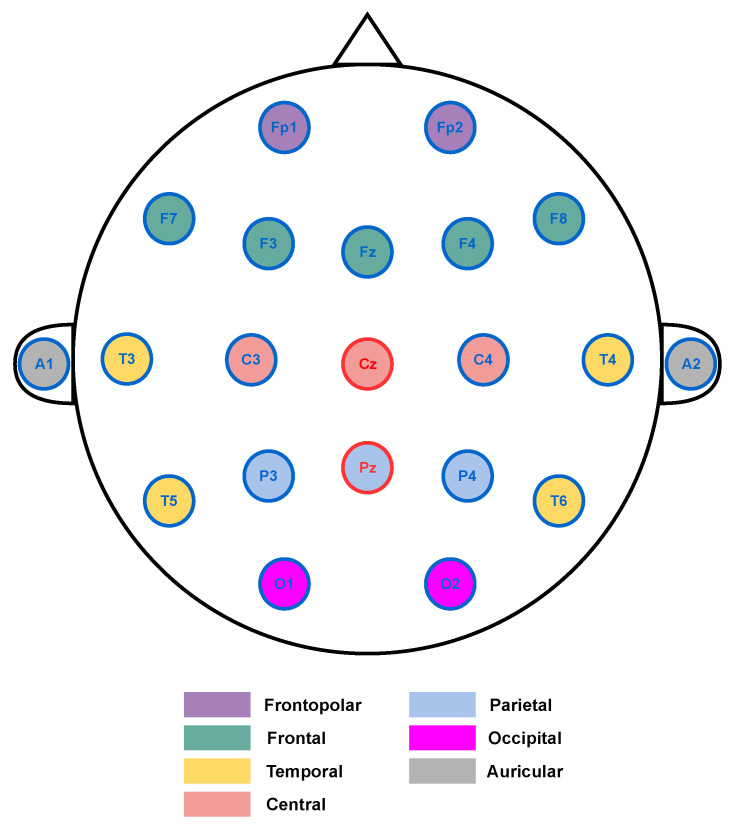
The international 10-20 system of electrode placement.

Figure 3 illustrates the international 10-20 electrode positioning system [67], with annotations to facilitate comparison with the patient data from the UBMC database. In the figure, the electrodes corresponding to regions affected by epilepsy in the patients documented in [66] are highlighted in blue. The channels that were not recorded, Cz and Pz, are indicated in red.

### 5.2. Experimental Setup

In this subsection, the experimental configuration of the presented proposal is detailed, in two phases.

#### 5.2.1. First Phase

Initially, after acquiring the database, it was decided to use the training and test files indicated in the database in .mat format. Data were segmented into 2 classes—ictal data (Complex Partial, Electrographic, and Video-detected with no visual change) and interictal data (no seizure). Subsequently, the EEG signals were pre-processed through z-score normalization for each channel. It is important to note that, after obtaining the data from the website, no additional data cleaning or artifact removal techniques were applied.

Subsequently, the EEG signals were segmented into 1 s time windows (each time window containing 500 samples). In the next phase, 13 features were extracted in the time domain for each of the 19 channels (Fp2, Fp1, F8, F4, Fz, F3, F7, A2, T4, C4, C3, T3, A1, T6, P4, P3, T5, O2, O1): amplitude, skewness, Activity, Complexity, Zero crossing, kurtosis, Energy, Maximum, mean, Median, Minimum, Mobility, and RMS. Thus, we generated 247 attributes (19 channels × 13 features). The mentioned features, Activity, Complexity, and Mobility, known as Hjorth parameters [68], were used specifically to represent EEG signals. The mathematical formulation of these features is described as follows:

Activity

(1) $$std^{2}=\frac{\sum_{i=1}^{N}{\left(x_{i}-\bar{x}\right)}^{2}}{N-1}$$

Amplitude

(2) $$amp=x_{max}-x_{min}$$

Complexity

(3) $$comp=\sqrt{\frac{mob(x\left(t\right)\left(\frac{dx}{dt}\right))}{mob(x(t))}}$$

Energy

(4) $$energy=\frac{1}{N}\sum_{i=1}^{N}x_{i}^{2}$$

Kurtosis

(5) $$k=\frac{Q_{3}-Q_{1}}{2(P_{90}-P_{10})}$$

Maximum value

(6) $$max=\max_{i=1}^{N}x_{i}$$

Mean

(7) $$\bar{x}=\frac{1}{N}\sum_{i=1}^{N}x_{i}$$

Median

median=(8)xN−12+1,for odd N;(9)12xN2+xN2+1,for even N

Minimum value

(10) $$min=\min_{i=1}^{N}x_{i}$$

Mobility

(11) $$mob=\sqrt{\frac{std^{2}\left(\frac{dx}{dt}\right)}{std^{2}}}$$

Root mean square

(12) $$rms=\sqrt{\frac{1}{N}\sum_{i=1}^{N}x_{i}^{2}}$$

Skewness

(13) $$s=\frac{\bar{x}-mode}{std}$$

Zero crossing

(14) $$zc=\left(K-1\right)-\frac{1}{2}\sum_{i=1}^{K}\left|\frac{x_{i}}{|x_{i}|}-\frac{x_{i+1}}{|x_{i+1}|}\right|$$

where $x_{i}$ is the value of each sample at position *i*. *N* is the vector dimension. *mode* is the mode of the vector. *std* is the standard deviation. $Q_{1}$ is quartile 1 of the vector. $Q_{3}$ is quartile 3 of the vector. $P_{90}$ is percentile 90 of the vector. $P_{10}$ is percentile 10 of the vector. For the calculation of the Median, it is necessary that the data array be sorted. For the calculation of Zero crossing, observe the sign changes between $x_{i}$ and $x_{i+1}$ in a series of *K* samples, excluding cases where $x_{i}=0$ or $x_{i+1}=0$.

Amplitude is a visual feature observed in EEG by experts. Epileptiform brain discharges exhibit high-amplitude deflections, typically in the order of hundreds of microvolts, whereas normal amplitudes range between 10 and 100 microvolts. Furthermore, high-amplitude rhythmic oscillations are used to define the onset and progression of epileptic seizures [3].

The features Energy and RMS contain important information about the measurement of amplitude. As seen from the formulas presented, Energy is the average of the sum of a signal squared, and RMS is the square root of Energy [69].

Second, Ref. [70] argues that through skewness, it is possible to identify distinct and clinically relevant patterns in sharp waves or spikes. Furthermore, they identified differences in the distribution of asymmetries between patients with unilateral abnormalities and patients with bilateral abnormalities, making it a useful tool for characterizing these types of epilepsy.

The study by [69] indicates that the Zero crossing feature is useful for EEG signal analysis, as the number of times the signal crosses zero is an indirect measure of the signal’s frequency. Therefore, high values of this feature indicate a higher frequency.

The study conducted by [69] highlights the utility of statistical parameters in distinguishing ictal and non-ictal patterns. Among these parameters, mean, Median, skewness, and kurtosis are mentioned. Additionally, the researchers note that the baseline of the EEG signal can be established based on the maximum and minimum values, which are also explored in this work.

The Hjorth parameters [68] are widely used as features in epilepsy detection studies [69]. Activity reflects signal power, indicating the spectral distribution of energy in the frequency domain. Mobility provides information about the average frequency change and variability in spectral distribution, while Complexity assesses the similarity of the signal to a sine wave, aiding in the identification of brain activity patterns [71].

The 247 attribute vectors were employed to train seven supervised learning classifiers: Decision Tree, k-Nearest Neighbor (kNN), Logistic Regression, Naive Bayes, Random Forest, eXtreme Gradient Boosting (XGBoost), and Support Vector Machine (SVM). The hyperparameters for these classifiers were optimized using GridSearchCV, with a specific focus on varying the number of estimators {5, 100, 300, 500} and the maximum depth {1, 3, 5, 10, 50}. Following the training and testing phase, the classifier demonstrating the highest accuracy on the test set was selected for use in subsequent phases.

The model achieving the highest accuracy was subsequently analyzed using SHAP, which facilitated the identification of attributes contributing most significantly to the classification. This tool enables the interpretation of the model’s decision-making process in predicting the data.

Upon application of SHAP, the top 20 features with the highest contribution to the model’s predictive performance were identified and visualized. Subsequently, a series of 20 models were trained and tested incrementally, each incorporating an additional SHAP-ranked feature. Specifically, model 1 was trained with only the most influential feature, model 2 with the first and second features, and so on, up to model 20, which included the first through twentieth features as ranked by SHAP.

This first phase aimed to reduce the number of attributes using models with relevant attributes incrementally.

#### 5.2.2. Second Phase

The 4 features and the 5 most recurrent channels presented in the first 20 attributes listed by SHAP in the previous phase were selected. This refined subset, encompassing 20 new attributes (5 channels × 4 features), was employed to train a new model. Subsequently, the SHAP analysis was reapplied to this model to rank the attributes according to their contribution to the predictive accuracy.

The classifier used in this phase was the one that obtained the best accuracy performance in the previous phase; however, once again GridSearchCV was used to define the hyperparameters (with the same variation mentioned earlier) using these new attributes.

Analogously to the first stage, a series of 20 models were trained, each incorporating a progressively increasing number of SHAP-ranked attributes. This was performed with the dual purpose of minimizing the attribute count and assessing the performance efficacy of the newly selected attribute set.

The metrics used to evaluate the performance of the classifiers were as follows: accuracy, precision, recall, and F1-score.

## 6. Results

In this section, the results obtained following the proposal presented in the methodology—in two phases—are presented, which constitutes the method that enables the reduction of input vectors in a humanly interpretable way.

### 6.1. First Phase

Table 2 presents the accuracy values obtained in training and testing using the five supervised classifiers (Decision Tree, kNN, Logistic Regression, Naive Bayes, Random Forest, XGBoost, and SVM) with the specified Hyperparameter. The result displayed in the table indicates that XGBoost, with a 97.43% accuracy in the test phase, is the most suitable to be used as a learning machine for the trained data, namely 247 attribute vectors (13 features and 19 channels).

**Figure 4:**
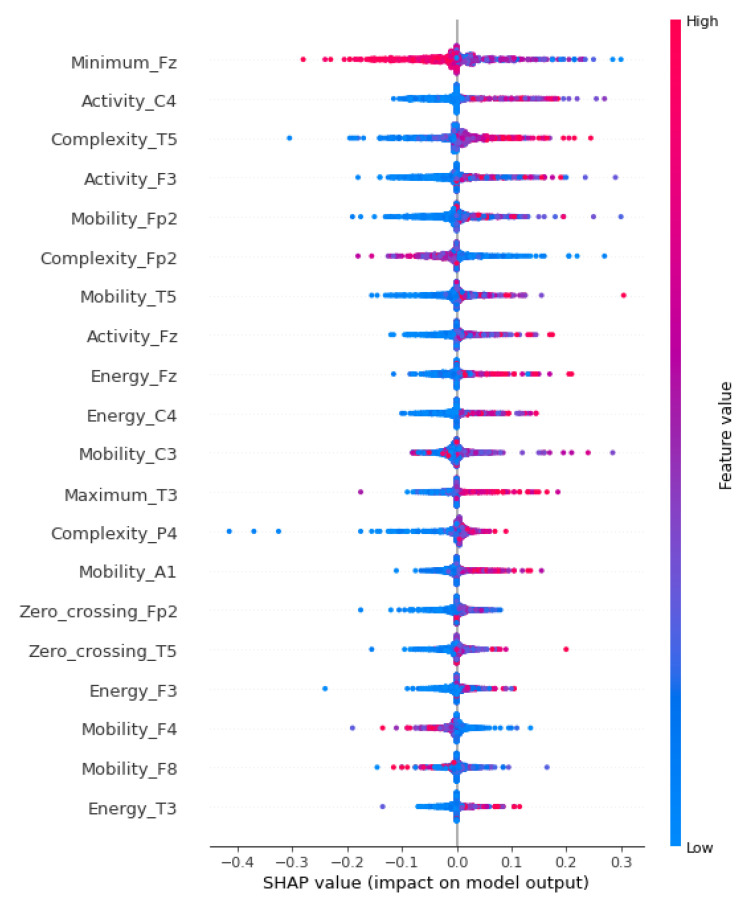
SHAP value—phase 1.

Figure 4 shows the graph of SHAP values for the first 20 attributes listed by SHAP (out of the 247 used in training with the XGBoost learning machine). The technique allows attributes to be listed in order of importance.

Observations reveal that specific attribute vectors contribute distinctly; for instance, elevated values in the Minimum_Fz vector (the Minimum feature calculated on the Fz channel) yield a negative impact on the prediction. Similarly, in the case of the second most influential attribute, Activity_C4, lower values are associated with a negative contribution.

The attributes were systematically organized for analysis: the first matrix incorporated solely the initial SHAP-ranked attribute (Minimum_Fz); the second matrix included both the first and second most influential attributes (Minimum_Fz and Activity_C4); and this sequential inclusion continued up to the twentieth matrix, which comprised the top 20 contributing attributes (Minimum_Fz, Activity_C4, Complexity_T5, Activity_F3, Mobility_Fp2, Complexity_Fp2, Mobility_T5, Activity_Fz, Energy_Fz, Energy_C4, Mobility_C3, Maximum_T3, Complexity_P4, Mobility_A1, Zero_crossing_Fp2, Zero_crossing_T5, Energy_F3, Mobility_F4, Mobility_F8, and Energy_T3). Each of these 20 matrices corresponded to one of the 20 trained models to which they were applied.

**Figure 5:**
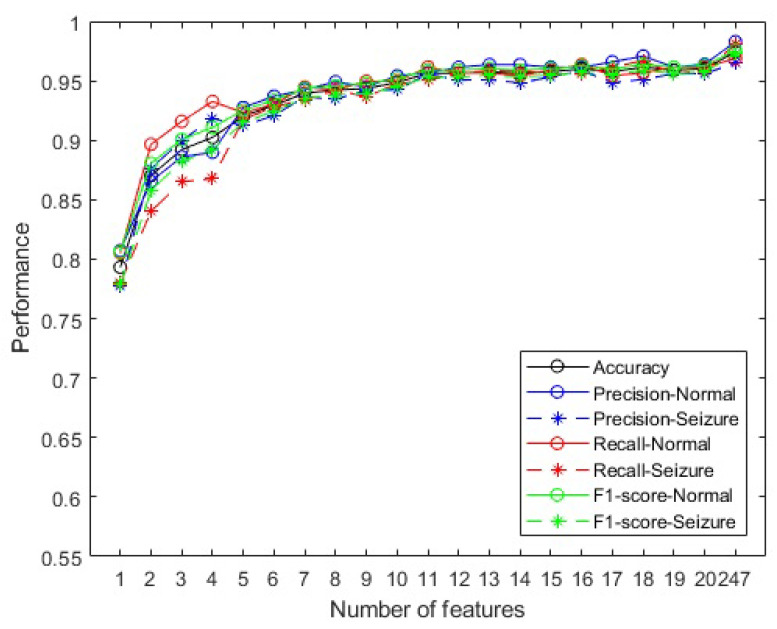
Phase 1—obtained performance.

Figure 5 presents the performance metrics—accuracy, precision, recall, and F1-score—of 21 models trained using XGBoost, comparing the results obtained with the top 20 SHAP-ranked attributes against those using the full set of 247 attributes.

### 6.2. Second Phase

In this second phase, the results obtained according to the steps presented in the second phase of the methodology are presented.

According to Figure 4, the four most common features were Activity, Complexity, Mobility, and Energy and the five most frequent channels were Fz, C4, T5, F3, and Fp2. In this way, the 20 new attributes are Activity_Fz, Activity_C4, Activity_T5, Activity_F3, Activity_Fp2, Complexity_Fz, Complexity_C4, Complexity_T5, Complexity_F3, Complexity_Fp2, Mobility_Fz, Mobility_C4, Mobility_T5, Mobility_F3, Mobility_Fp2, Energy_Fz, Energy_C4, Energy_T5, Energy_F3, and Energy_Fp2.

Table 3 showcases the performance metrics of the XGBoost classifier, which was configured with hyperparameters set to a maximum depth of 50 and 500 estimators, utilizing the previously identified 20 new attributes.

**Figure 6:**
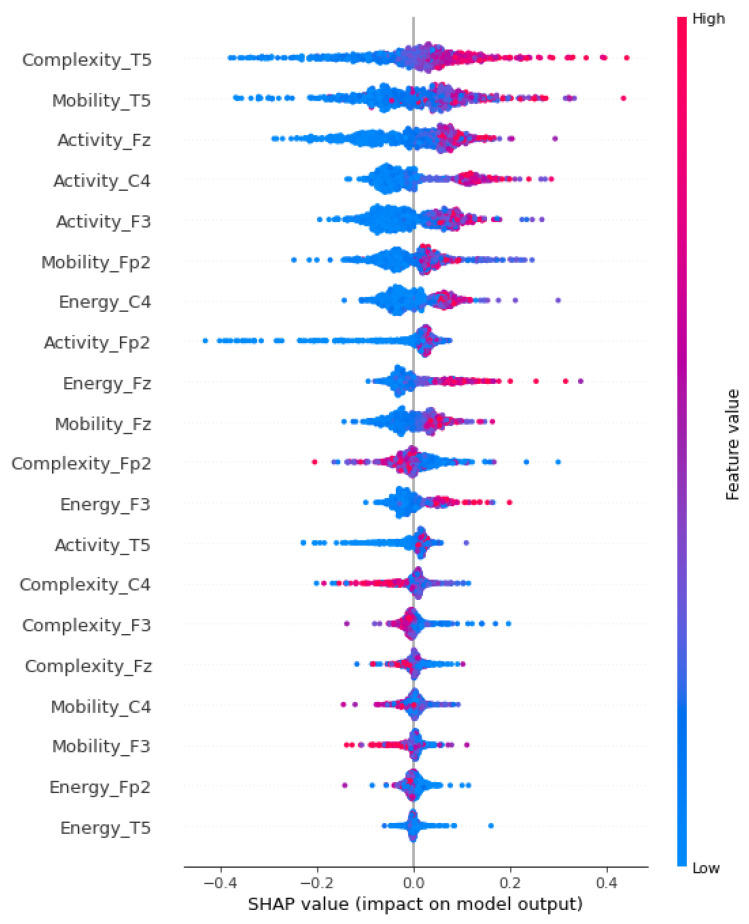
SHAP value—phase 2.

Figure 6 presents a graph with the contribution of the SHAP values of the 20 most recurrent attributes selected.

It is possible to observe the contributions of each attribute vector. For example, low SHAP values in the first five attributes have a negative contribution to the prediction; similarly, the positive contribution is more present for high SHAP values.

Drawing from Figure 6, the attributes for constructing the 20 matrices were methodically organized in a manner akin to the approach taken in the preceding phase. Consequently, the initial matrix exclusively encompasses the attribute with the foremost predictive contribution, which is Activity_Fz. This sequence continues incrementally until the composition of the twentieth matrix, which incorporates all 20 attributes as ranked by SHAP (Activity_Fz, Activity_C4, Activity_T5, Activity_F3, Activity_Fp2, Complexity_Fz, Complexity_C4, Complexity_T5, Complexity_F3, Complexity_Fp2, Mobility_Fz, Mobility_C4, Mobility_T5, Mobility_F3, Mobility_Fp2, Energy_Fz, Energy_C4, Energy_T5, Energy_F3, and Energy_Fp2). Each of these matrices was subsequently applied to its corresponding model within the cohort of 20 trained models.

**Figure 7:**
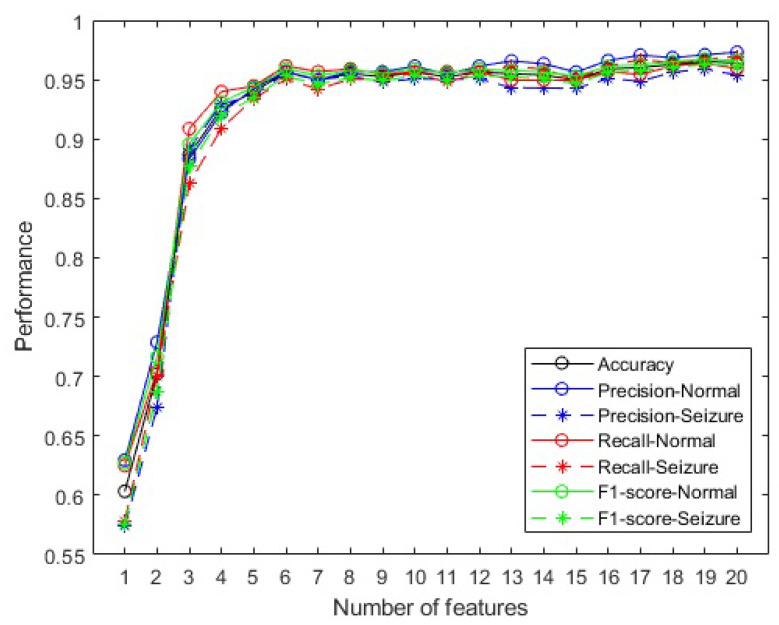
Phase 2—performance.

Figure 7 contains the performance of 20 models trained for accuracy, precision, recall, and F1-score using XGBoost with the 20 attributes listed by SHAP.

The highest accuracy achieved in this phase occurred with 19 attributes (96.53%), and with 6 it was already possible to obtain an accuracy greater than 95% (95.93%).

**Figure 8:**
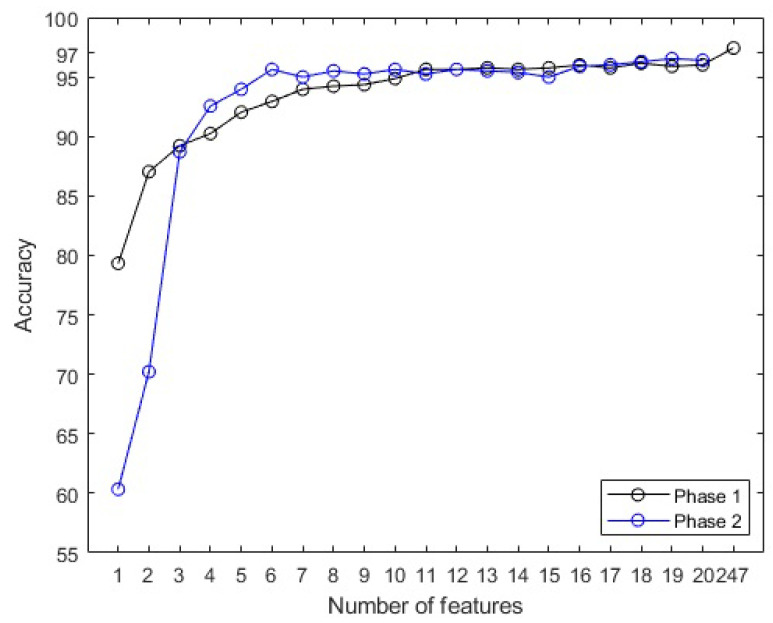
Phase 1 and 2 accuracies (%).

Figure 8 presents the accuracy obtained in the two distinct phases of model training. In the first phase, employing a single attribute from the full set of 247, the model attained an accuracy of 79.33%. Conversely, in the second phase, when using only the top SHAP-ranked attributes from a reduced set of 20, the accuracy diminished to 60.33%. This reduction is attributable to the fact that the initial selection was made from a larger pool of attributes, whereas the second was constrained to a more limited subset.

Table 4 shows the comparison of the obtained results. It represents a brief description of the methodology and the performance according to the metrics used in each experiment.

## 7. Discussion

A method for reducing channels and features using an XAI technique was introduced in the creation of an optimized model for epileptic seizure detection. The ensuing section delineates the outcomes of this approach.

Initially, the XGBoost algorithm emerged as the classifier of choice, demonstrating a high accuracy rate exceeding 97%, albeit dependent on a full set of 247 features. Subsequent application of the SHAP methodology to the trained model and corresponding test data facilitated the generation of streamlined models, which were then organized according to the predictive significance of their features.

The data depicted in Figure 5 reveal that the model encompassing 18 attributes attains the highest test accuracy at 96.15%; notwithstanding, a marginal disparity persists between the precision, recall, and F1-score metrics across both classes. These metrics attain stability upon expanding to a model with 20 attributes, evidenced by a 96.02% accuracy rate. This outcome is in close concordance with the results delineated in Table 2, where the XGBoost classifier realizes a 97.43% accuracy. Such findings corroborate the feasibility of achieving comparable model efficacy with a substantial reduction in input vectors, exceeding 91%, in this initial phase.

It is noteworthy that the model, with a configuration of merely 11 attributes, attained an accuracy of 95.63%, a figure that stands numerically on par with the accuracies reported in studies employing deep learning models [12,15].

In the subsequent phase of the study, a pattern of recurrence was noted among specific channels and features within the top 20 SHAP-identified attributes. This recurrence guided the synthesis of a new set of models, derived from the amalgamation of these frequently appearing channels and features.

Looking at Table 4, it can be noted that in the first phase, with 11 attributes (Minimum_Fz, Activity_C4, Complexity_T5, Activity_F3, Mobility_Fp2, Complexity_Fp2, Mobility_T5, Activity_Fz, Energy_Fz, Energy_C4, Mobility_C3) and using of six channels (Fz, C4, T5, F3, Fp2, and C3), it was possible to achieve satisfactory performance for binary classification. The second phase proved to be relevant by reducing the number of input vectors to six (Activity_Fz, Activity_C4, Activity_T5, Activity_F3, Activity_Fp2, Complexity_Fz) and the number of channels to five (Fz, C4, T5, F3, and Fp2), still achieving similar performance as the previous phase (above 95% accuracy).

It is relevant to relate the five channels selected in phase 2 (Fz, C4, T5, F3, and Fp2) to the regions where patients’ focal points are located in the dataset. The selected channels, C4 and Fp2, correspond to focal points in some of the patients. Channel T5 is present in the following focal regions: posterior temporal and left hemisphere. Channel F3 is present in the frontotemporal focal region and left hemisphere, and channel Fz is present in the frontotemporal focal region. Therefore, the selected channels are spatially aligned with the focal points of patients’ clinical events observed in EEG. This indicates that the method selects channels that are consistent with the location of clinical events in epileptic seizures.

Numerically comparing the two phases in terms of performance, as depicted in Figure 8, it becomes apparent that the second phase of the methodology excels. Specifically, within the range of three to nine attributes (x-axis), the accuracy attains higher values, which is advantageous for employing the model with a minimal number of input vectors while still achieving enhanced performance.

Observing the aspects of feature selections, in the first phase, Figure 4 highlights the most significant feature, representing the minimum value (located in channel Fz), as a key element in discriminating the data. This observation aligns with the concept mentioned in [69], emphasizing the role of the minimum value in defining a reference point for distinguishing non-ictal data from ictal signals. The chart in Figure 5 corroborates this importance, showing that this attribute alone can achieve a remarkable performance of approximately 80% in various binary classification metrics.

In the second phase, the most common attributes—Activity, Complexity, Mobility, and Energy—are often used together in related works, as cited in the study in [69]. Although 13 attributes were initially selected in the first phase, with detailed justifications, it is noteworthy that these 4 selected attributes are capable of effectively discriminating classes for specific channels. This fact demonstrates that it is feasible to reduce the number of features and channels, even in generalist models.

Some potential biases need to be considered in the interpretation of the results: the UBMC database is limited as it only includes six patients and lacks data for all types of epilepsy. Therefore, the selected channels may be specific to the patients in the database, and consequently, the computed features may not be universally applicable, even in more generalized models. Additionally, the selection based on SHAP may be influenced by the specific dataset. Hence, generalizing the results requires caution and validation on additional datasets that utilize the same data segments, such as TUH and CHB-MIT.

It is also worth noting that the SHAP value method assumes that the model features are independent of each other [26]. This suggests that SHAP may not fully capture the interactions between the features. This limitation is not favorable for this application since the sensors are spatially distributed on the scalp, where epileptic discharges are measured through the channels.

In this way, it was possible to observe that simpler classification models can be efficient in the identification of epileptic seizures. Additionally, the explainable method of feature and channel selection allowed for reducing computational effort while maintaining high performance. This method, by not transforming the dataset, allowed us to verify that the chosen channels are located in focal areas of epileptic seizures. This encourages further study with customized models, which may further reduce the number of channels and confirm if the focal point will always be the best location for extracting data for epileptic seizure detection.

## 8. Conclusions

In light of these observations, the proposed approach has the potential to significantly streamline computational processes by reducing both the quantity of input vectors and the complexity of the classification models. Moreover, it may contribute to the simplification of signal acquisition hardware through a minimized electrode array, all the while sustaining a high standard of accuracy in distinguishing between interictal and ictal EEG data.

Considering that the methods used in the literature have a large number of input vectors [10,11,18] (which also requires a greater number of samples for training) and make use of more robust machines such as DL [13,14], the proposed approach simplifies the solution by delivering a numerically equivalent performance, employing temporal feature extraction, using simpler classifiers (XGBoost), and reducing the dimensionality through an explainable technique (SHAP), enabling model interpretability.

Utilizing a database with detailed focal point locations enabled confirmation that the model’s most significant channels are spatially proximate to the patients’ focal zones. This outcome fosters the impetus for subsequent research to investigate personalized models, which could validate the hypothesis that optimal channels for seizure detection predominantly reside within these focal areas. Such advancements could further curtail the requisite number of channels and features, thereby facilitating the development of mobile applications.

Although a satisfactory performance was achieved, it is necessary to replicate the method for a database with a larger number of patients that contains information about the location of the seizures. Creating a universal generalist model is a challenge, as it depends on a representative database. However, this study encourages the application of the method in personalized models, where it is expected to further reduce the number of channels and features.

## Figures and Tables

**Table 1:** Comparative studies with binary classification (interictal vs. ictal) of EEG signals.

Methodology	Performance
Authors: [7]	Accuracy: 0.91
Database: TUH	F1-score: 0.91
Domain: time and frequency	AUC: 0.95
Classifier: Random Forest	
Dimensionality reduction: None	
Channels: 19	
Features: 19	
Attributes: 19 × 19 = 361	
Authors: [8]	Accuracy: 0.93
Database: TUH	Precision: 0.94
Domain: time and frequency	Recall: 0.94
Classifier: SVM	Specificity: 0.91
Dimensionality reduction: None	F1-score: 0.94
Channels: 21	
Features: 13	
Attributes: 21 × 13 = 273	
Authors: [9]	Precision: 0.73
Database: TUH	Recall: 0.79
Domain: frequency	F1-score: 0.78
Classifier: Logistic Regression	
Dimensionality reduction: PCA	
Channels: 21	
Features: 5	
Attributes: 7	
Authors: [10]	F1-score: 0.84
Database: TUH	
Domain: frequency	
Classifier: LightGBM	
Dimensionality reduction: None	
Channels: 21	
Features: 96	
Attributes: 2016	
Authors: [11]	Accuracy: 0.88
Database: TUH	Recall: 0.83
Domain: frequency	Specificity: 0.91
Classifier: CatBoost	
Dimensionality reduction: None	
Channels: 21	
Features: 96	
Attributes: 21 × 96 = 2016	
Authors: [12]	(Interictal; Ictal)
Database: CHB-MIT	Accuracy: 0.99; 0.99
Domain: time and frequency	Precision: 1; 0.98
Classifier: SVM	Recall: 1; 1
Dimensionality reduction: None	F1-score: 0.99; 0.99
Channels: 22	
Features: 18	
Attributes: 22 × 18 = 396	
Authors: [18]	Accuracy: 0.99
Database: CHB-MIT	Recall: 0.99
Domain: time and frequency	Specificity: 0.83
Classifier: Random Forest	
Dimensionality reduction: None	
Channels: 20	
Features: 8	
Attributes: 1328	
Authors: [19]	Precision: 0.47
Database: CHB-MIT	Recall: 0.81
Domain: frequency	F1-score: 0.56
Classifier: KNN	
Dimensionality reduction: t-SNE	
Channels: 21	
Features: 1	
Attributes: 2	

**Table 2:** Classification result using 247 features.

Classifier	Hyperparameter	Training Accuracy Mean	Test Accuracy
Decision Tree	Max depth: 15 Min samples split: 2	92.34%	93.07%
kNN	Num neighbors: 1	93.68%	93.58%
Logistic Regression	Default	84.69%	84.21%
Naive Bayes	Gaussian	79.46%	81.00%
Random Forest	Max depth: 50 Num estimators: 150	96.62%	96.28%
XGBoost	Max depth: 5 Num estimators: 300	97.83%	97.43%
SVM	RBF	84.74%	87.29%

**Table 3:** Performance of XGBoost with the 20 new features.

Train Acc Mean	Test Acc	Real Class	Confusion Matrix		Precision	Recall	F1-Score
96.20	96.40	Interictal	399	17	97.32	95.91	96.61
		Ictal	11	352	95.39	96.97	96.17

**Table 4:** Comparison of the results obtained in the two phases.

Methodology	Performance
Phase: 1	Accuracy: 97.43
Dimensionality reduction: No	(Interictal; Ictal)
Channels: 19	Precision: 98.29; 96.48
Features: 13	Recall: 96.88; 98.07
Attributes: 19 × 13 = 247	F1-score: 97.58; 97.27
Phase: 1	
Dimensionality reduction: SHAP	
Channels: 11 (Fz, C4, T5, F3, Fp2,	Accuracy: 96.02
C3, T3, P4, A1, F4, F8)	(Interictal; Ictal)
Features: 7 (Minimum, Activity,	Precision: 96.39; 95.60
Complexity, Mobility, Energy,	Recall: 96.15; 95.87
Maximum, Zero crossing)	F1-score: 96.27; 95.74
Attributes: 20	
Phase: 1	
Dimensionality reduction: SHAP	Accuracy: 95.64
Channels: 6 (Fz, C4, T5, F3, Fp2, C3)	(Interictal; Ictal)
Features: 5 (Minimum, Activity,	Precision: 95.69; 95.57
Complexity, Mobility, Energy)	Recall: 96.15; 95.04
Attributes: 11	F1-score: 95.92; 95.30
Phase: 2	
Dimensionality reduction: SHAP	Accuracy: 96.41
Channels: 5 (Fz, C4, T5, F3, Fp2)	(Interictal; Ictal)
Features: 4 (Activity, Complexity,	Precision: 97.32; 95.39
Mobility, Energy)	Recall: 95.91; 96.97
Attributes: 20	F1-score: 96.61; 96.17
Phase: 2	Accuracy: 95.64
Dimensionality reduction: SHAP	(Interictal; Ictal)
Channels: 5 (Fz, C4, T5, F3, Fp2)	Precision: 95.69; 95.57
Features: 2 (Activity, Complexity)	Recall: 96.15; 95.04
Attributes: 6	F1-score: 95.92; 95.30

## Data Availability

The data presented in this study are openly available in Mendeley Data, titled “Epileptic EEG Dataset” by Nasreddine, Wassim (2021), V1, available at doi: https://doi.org/10.17632/5pc2j46cbc.1.

## Abbreviations

The following abbreviations are used in this manuscript:

CHB-MIT	Children’s Hospital Boston at the Massachusetts Institute of Technology.
DL	Deep Learning.
DWT	Discrete Wavelet Transform.
EEG	Electroencephalography.
XAI	Explainable Artificial Intelligence.
XGBoost	eXtreme Gradient Boosting.
FFT	Fast Fourier Transform.
kNN	k-Nearest Neighbor.
ML	Machine Learning.
PCA	Principal Component Analysis.
SHAP	SHapley Additive exPlanations.
SVM	Support Vector Machine.
t-SNE	t-Distributed Stochastic Neighbor Embedding.
TUH	Temple University Hospital.
UBMC	University of Beirut Medical Center.
